# The Invisible Work of Personal Health Information Management Among People With Multiple Chronic Conditions: Qualitative Interview Study Among Patients and Providers

**DOI:** 10.2196/jmir.4381

**Published:** 2015-06-04

**Authors:** Jessica S Ancker, Holly O Witteman, Baria Hafeez, Thierry Provencher, Mary Van de Graaf, Esther Wei

**Affiliations:** ^1^ Division of Health Informatics Department of Healthcare Policy and Research Weill Cornell Medical College New York, NY United States; ^2^ Department of Family and Emergency Medicine Faculty of Medicine Laval University Québec City, QC Canada; ^3^ Office of Education and Continuing Professional Development Faculty of Medicine Laval University Québec City, QC Canada; ^4^ Research Centre of the CHU de Québec Québec City, QC Canada; ^5^ Department of Medicine Weill Cornell Medical College New York, NY United States

**Keywords:** consumer health information technology, electronic medical records, electronic patient portals, personal health records (PHRs), chronic disease, qualitative research, diabetes, information management

## Abstract

**Background:**

A critical problem for patients with chronic conditions who see multiple health care providers is incomplete or inaccurate information, which can contribute to lack of care coordination, low quality of care, and medical errors.

**Objective:**

As part of a larger project on applications of consumer health information technology (HIT) and barriers to its use, we conducted a semistructured interview study with patients with multiple chronic conditions (MCC) with the objective of exploring their role in managing their personal health information.

**Methods:**

Semistructured interviews were conducted with patients and providers. Patients were eligible if they had multiple chronic conditions and were in regular care with one of two medical organizations in New York City; health care providers were eligible if they had experience caring for patients with multiple chronic conditions. Analysis was conducted from a grounded theory perspective, and recruitment was concluded when saturation was achieved.

**Results:**

A total of 22 patients and 7 providers were interviewed; patients had an average of 3.5 (SD 1.5) chronic conditions and reported having regular relationships with an average of 5 providers. Four major themes arose: (1) Responsibility for managing medical information: some patients perceived information management and sharing as the responsibility of health care providers; others—particularly those who had had bad experiences in the past—took primary responsibility for information sharing; (2) What information should be shared: although privacy concerns did influence some patients’ perceptions of sharing of medical data, decisions about what to share were also heavily influenced by their understanding of health and disease and by the degree to which they understood the health care system; (3) Methods and tools varied: those patients who did take an active role in managing their records used a variety of electronic tools, paper tools, and memory; and (4) Information management as invisible work: managing transfers of medical information to solve problems was a tremendous amount of work that was largely unrecognized by the medical establishment.

**Conclusions:**

We conclude that personal health information management should be recognized as an additional burden that MCC places upon patients. Effective structural solutions for information sharing, whether institutional ones such as care management or technological ones such as electronic health information exchange, are likely not only to improve the quality of information shared but reduce the burden on patients already weighed down by MCC.

## Introduction

Some 90 million individuals in the United States are affected by more than one chronic disease simultaneously, and the number of people with “multiple chronic conditions” (MCC) continues to grow as the population ages [1]. The designation of MCC is a broad one that has been defined by the US Department of Health and Human Services as any combination of conditions that last at least one year and that require ongoing medical attention or limit activities of daily living [1]. Individuals can be described as having MCC if they have two or more of any physical or mental conditions (heart disease, depression, anxiety, diabetes, asthma, arthritis, HIV, chronic obstructive pulmonary disorder, chronic pain, etc). Medical care for individuals with MCC is challenging as the evidence base about specific combinations of conditions may be weak or absent, and the therapies and management strategies for a particular condition might be contraindicated by another condition [1].

Dealing with the health care system is potentially very challenging for patients with MCC, as they typically consult more doctors and have more medical appointments than patients with single conditions [1]. One critical problem for patients who see multiple health care providers is the issue of communication among those providers. Clinicians and policymakers have long recognized that critical patient data is often missing at clinical encounters even in medically straightforward situations [2], and that the chances of missing data increase with care transitions [3,4]. Such missing data contributes to lack of care coordination, low quality of care, and medical errors [2-6].

Potential health information technology (HIT) solutions have been focused primarily on facilitating provider-to-provider information sharing, including interoperable electronic health records (EHRs) and health information exchange (HIE) systems [7-9]. However, in addition, a number of consumer technologies offer patients the opportunity to transfer their own records across care settings, a process known as consumer-mediated or patient-mediated HIE [10]. These include patient-controlled personal health records (PHRs), electronic patient portals managed by health care organizations, and Blue Button functionalities that allow patients to export medical record information for personal use [11-16]. Consumer surveys frequently find strong public support for the concept of patient-mediated HIE [17-20]. Yet concerns have been expressed about whether all patients will be sufficiently engaged or informed to serve as stewards of their own data, whether patients might suppress or alter sensitive information [10], and whether socioeconomically disadvantaged and elderly patients will have adequate computer access or skills to use these technologies [21]. Recent data shows that patient use of portals and PHRs is beginning to climb, but these tools are still reaching only a minority of the public [14].

From the patient perspective, the tasks involved in collecting and managing personal medical information have been called “personal health information management” *(*PHIM) [22-25]. PHIM encompasses a variety of activities conducted largely outside the medical encounter: examples include tracking health data, seeking information, and organizing it [22], creating personal histories, and planning medical activities [23], and providing records to doctors [26]. As these are all effortful, directed activities to attain goals, it is appropriate to recognize them as *work* [22,27-29]. Most PHIM activities fall in the category of “illness work”, that is, the activities involved with managing an illness, such as taking medicines, getting information, and using technologies such as blood glucose meters [27-29]. Other PHIM activities constitute “articulation work”, in other words, the planning and managing tasks that allow people to complete other types of work, whether illness work or everyday life work [27,28]. Articulation work might include such essential tasks as keeping a family calendar or organizing transportation to medical appointments.

A rich PHIM literature is developing. Some work has focused on healthy individuals and families [22,23,26,30] and on computer-literate participants [26]. Another body of work is developing on patients with cancer [31-34]. As part of a broader project on potential applications of consumer HIT and barriers to its use, we sought to explore PHIM conducted by patients with MCC, whose long-term complex medical situations would be expected to result in heavy demands for information management. Our qualitative study focused on the management of medical information and medical records. Our research questions were: How do patients with MCC manage their medical records and medical information sharing with medical providers? How do they perceive their role in managing their medical information? Management of information was defined broadly to include information transfers across the patient’s network of current providers as well as during care transitions from one provider to another.

## Methods

### Participants

As described in the companion piece to this paper [35], we recruited adult English-speaking patients with MCC, as well as health care providers with experience providing care for patients with MCC. Patients and providers were recruited independently from the same settings but not specifically to represent patient-provider pairs. One researcher (JSA) also attended six 90-minute sessions of a diabetes education support group in order to triangulate themes arising from patient interviews. The diabetes group was chosen because diabetes is prevalent among patients with MCC, because the majority of the patients in the diabetes group had at least one comorbid condition, and also because of availability (we found no local group education programs focusing on MCC).

The primary focus of the study was on the patient perspective. Provider interviews were used to triangulate themes arising in patient interviews, explore situations in which provider perspectives contrasted with patient perspectives, and fact-check medical concepts.

### Settings

Participants were recruited from Weill Cornell Physicians (a multispecialty academic medical practice in Manhattan), New York-Presbyterian Hospital (the largest academic hospital in Manhattan), and the Institute for Family Health (a federally qualified health center serving New York City). We distributed promotional flyers at the three institutions, and also elicited referrals from physicians and nurse practitioners at outpatient clinics in internal medicine and endocrinology. Patient interviews were conducted in conference rooms or spare offices at the three locations, usually immediately before or after a clinical visit. Provider interviews were conducted in provider offices.

### Interview Methods

We developed a semistructured interview instrument about PHIM (the focus of the current manuscript) as well as the related topic of personal health information tracking (reported elsewhere [35]). The interview guide included questions about: (1) how patients perceived their level of knowledge about their medical conditions, (2) times they had looked up or done research on health topics, (3) whether they tracked or logged information about their personal health or their medical care (probe questions asked about types of information such as medications, diet and exercise, personal medical data such as blood glucose, records of doctor’s visits or of surgical procedures, etc), and (4) information or documents they typically brought to share with their doctor or nurse, including information that they brought when moving from one doctor or medical center to another. A follow-up probe question asked if they had ever looked at their medical chart as a Web portal, via a phone, or as a paper record. Interviews were conducted in person, audiorecorded, and professionally transcribed. The interviewer (JSA) also took field notes and photographed artifacts or documents such as log sheets used to record blood glucose values.

### Analysis Methods

Qualitative analysis was conducted by our multidisciplinary team, which included members with training in journalism, public health, informatics, psychology, human factors, nursing, and diabetes education. Two of the researchers (HOW and EW) also brought personal experience of chronic disease or multiple chronic disease. Following Strauss and Corbin’s grounded theory methods [36], we conducted open coding (allowing codes to emerge from the data), axial coding (identifying relationships), and selective coding (developing underlying themes and theory). Each transcript and photograph was reviewed by at least two researchers (the 1st author and one or more additional team members), who coded independently and then met to reach consensus. Interrater reliability was not calculated as coding was finalized during consensus meetings. A total of 47 open codes were developed, which were grouped via axial coding into six broader concepts before the final themes were identified.

Analysis was conducted concurrently with recruitment, which was halted when saturation was achieved (ie, no new concepts were arising from new interviews) [37]. Fewer providers were recruited than patients because provider perspectives proved more homogeneous in the analysis.

Member checking [38] was conducted by (1) discussing emergent concepts and themes with new informants, and by (2) presenting the final list of themes in a 90-minute session of the diabetes education group. Two members of the diabetes education group had previously participated in an individual interview as part of the study. During the member check, the themes appeared to resonate strongly with the participants, many of whom offered additional anecdotes and personal experiences. In the post-member check meeting, the researchers concluded that all of the new comments and anecdotes were congruent with the existing themes.

### Ethics Approval

This study was approved by the Institutional Review Boards of Weill Cornell Medical College and the Institute for Family Health. All interview participants gave written informed consent. Members of the diabetes education group provided oral consent at each session that the researcher attended. During individual interviews, permission was asked to take photographs of patient artifacts that excluded identifying information; participants reviewed each photograph as it was taken and decided whether it would be deleted or saved.

## Results

### Participants

Interviews were conducted with 22 patients and 7 health care providers. Slightly more than half of the patients (13/22, 59%,) had a relationship with one of the providers who was interviewed, and the rest did not. Conversely, 4 of the 7 providers had patients who were included in the study.

Patients had an average of 3.5 (SD 1.5) chronic conditions, including type 2 diabetes, hypertension, heart disease, chronic pain, depression, asthma, HIV, and hepatitis C. Several reported taking anticoagulants, although they did not all explain what condition they had. Participants mentioned regular relationships with an average of 5 different providers, including primary care physicians or nurse practitioners; medical and surgical specialists; allied health providers including physical therapists, dietitians, and diabetes educators; pharmacists; and dentists or oral surgeons. In addition, many of the patients had had recent visits to an emergency department or urgent care center for urgent conditions, which included diverticulitis, flu, appendicitis, burns, and other physical injuries.

The patient sample was half men (11/22, 50%) and half women (11/22, 50%); 7 of the 22 patients (32%) were black. The average age was 64 years (range 37-89). Two-thirds (15/22, 68%) were not currently married; 8 of 22 (36%) used English as a second language. One-third (7/22, 32%) had Medicare (US public insurance for those over age 65), one-third (7/22, 32%) had Medicaid (US public insurance for low income individuals), and the remainder had commercial insurance (8/22, 36%).

The health care providers were 2 nurse practitioners, 2 internists, 2 family medicine physicians, and an emergency medicine physician (4 women and 3 men).

Major themes pertaining to PHIM are summarized in Table 1 and presented in detail below.

**Table 1:** Major themes in Personal Health Information Management.

Themes	Summary	Representative quotes
A. Responsibility for managing medical information across organizational settings	Some patients perceive medical records management as the health care system’s responsibility, whereas others perceive it as their own.	“[The doctors] are supposed to have all the information. They’re supposed to look it up.”
B. What medical information should be shared?	Patients make frequent judgments about what data is relevant to their health and therefore should be shared or reported.	“The things that [the dermatologists] were doing really wasn’t, you know, something that [my primary care doctor] needed to know.”
C. Methods, tools, artifacts	Patients who took an active role in managing their records used electronic tools, paper, and memory	“I keep it in my head... I know the dosage, the day, for what is this medicine and how many times I [take it] daily.”
D. Managing medical information as “invisible work”	Managing transfers of medical information to solve problems such as health insurance denials is a tremendous amount of work that largely goes unrecognized.	“It’s hard enough when you’re healthy and you’re with it, and you’re feeling good… When you’re not feeling well at all, it’s difficult.”

### Theme A: Responsibility For Sharing Medical Information Across Organizations

We found a range of opinions about who—patients, providers, or both—had primary responsibility for sharing medical information and records.

#### Patients’ Responsibility

Many of the people with MCC felt strong responsibility for sharing their medical information and records across their networks of providers. “It’s up to you [to keep track of that information], really,” said one. Some of the patients with this perspective had developed their approach because of previous negative experiences in which important information from one provider had failed to reach another provider. These individuals often recorded or memorized their own information, brought documents from one provider to another, or requested transferal of lab results, records, and imaging studies from one provider to another. It was very common for patients to maintain a written, printed, or memorized medication list because they knew that they might be asked to provide it to a new doctor or to emergency room staff. Sometimes, the responsibility was assumed by a family member (often a female family member such as a patient’s wife or an elderly patient’s adult daughter).

#### Responsibility of the Medical System

However, other patients perceived medical information management to be primarily the responsibility of the health care system. “They’re supposed to have all the information. They’re supposed to look it up,” said one individual, who seemed surprised to be asked about it. Patients sometimes expressed a preference for going to a hospital where “they know me” because of previous records. Two even mentioned the shared electronic health record as a reason why they sought primary and specialty care within the same institution. Even a few patients who did not themselves use computers knew that EHRs were being used to capture and share their information: one described his chart as being “on the terminal”’ and another called it “the modern thing”. Providers confirmed that some patients did not take a very active role in informing their providers about their other ongoing relationships with physicians or their previous records.

#### Confidence and Trust

Among the patients, beliefs about responsibility for information management appeared to be closely linked to feelings of confidence in doctors and health care organizations. One patient said it was important to ask questions and collect records because, “I believe you have to keep the doctors honest.” Conversely, another patient, when asked whether he would be interested in accessing his medical record via the portal, said, “Why should I ask for it? I’m being seen on a regular basis for everything.”

#### Providers’ Pragmatism

Providers were unanimous that they needed easy access to their patients’ information from other institutions in order to make the best decisions about their care. They were pragmatic, saying that any way of getting information was preferable to not having information. Methods of obtaining previous information and records about a patient included searching the institution’s own records, interviewing the patient, interviewing the patient’s family, asking the patient to bring copies of records from previous institutions, and calling other physicians and health care organizations to obtain oral reports or faxes. Only one provider reported having a patient who logged in to an electronic patient portal to retrieve information from a previous institution. Despite patients’ confidence in computers, the providers recognized that data in electronic format was not necessarily shareable. The providers interviewed relied heavily upon EHRs within their organizations but also complained about lack of interoperability between different health care organizations and sometimes even different divisions within the same organization. It also was common for them to express frustration with patients who could not clearly report their own history. In some cases, providers suspected patients were trying to conceal information. “Whether it’s they don’t want us to contact [the previous doctor] or they really just don’t remember is an issue.”

### Theme B: What Medical Information Should Be Shared?

Those patients who were instrumental in information sharing across physicians and health care organizations made judgments about what information was important to share with whom. These judgments were linked to their understanding of their medical conditions, their understanding of the health care system, and privacy issues.

#### Understanding of Disease

Patients generally wanted to share information relevant to their care. However, patients’ decisions about which information was relevant were influenced by concepts of health, disease, and relationships between diseases. For example, one patient judged that there was no need for information sharing between a dermatologist treating a scar and her other physicians: “[It] really wasn’t, you know, something that they needed to know.” Almost all the patients seemed familiar with the concept of medication interactions as the justification for providing their complete medication list to all of the physicians that they saw. Yet most, when asked, said they did not tell their medical providers about herbal treatments, dietary supplements, or dental visits, and many said dentists had never asked about their medical conditions.

Providers also talked about patients’ selective reporting of information, generally ascribing it to limited health literacy. For example, physicians talked about needing to instruct a patient to obtain previous laboratory results or medical records. According to one physician’s anecdote, a patient failed to report partial loss of vision in one eye while being examined for possible multiple sclerosis; the physician believed the omission was motivated by denial, but an alternative explanation is that the patient had no idea it might be relevant.

#### Privacy Concerns

In only a few cases, we encountered patients who were concerned that medical information would be used against them. A woman with a previous psychiatric diagnosis believed her history had been misused by ambulance personnel who “put my name in the computer” and diverted her to psychiatric care instead of the medical emergency care she was seeking. Another individual was concerned about how doctors interpreted the history of sexually transmitted infection in his medical record. One woman was strongly motivated to conceal her diabetes from her insurer because she was concerned the company would raise her premiums.

#### Understanding of the Health Care System

In addition, patients’ decisions about sharing medical information were shaped by their experience with and understanding of health care systems. Patients with diabetes who saw multiple health care providers generally learned that they would be asked about their hemoglobin A1c results by all of them. One woman explained why she knew to bring her medication list to a hospital appointment: “Well, I’ve been in the hospital before, or even another doctor’s appointment, ‘what medicines are you taking?’ And they always want you to fill it out again.” One woman explained that she didn’t think that dental information was relevant to doctors because she had never had a doctor ask about it. A large number of the patients recognized that their pharmacist was likely to check their medication list for potential interactions (or “clashes”, in the words of one woman). Misconceptions about the health care system could also play a large role in patient decision-making. One patient said that she did not need to bring x-rays from one hospital to another nearby one because the doctors could see each other’s computer systems. (A post-interview fact check showed this was not the case.) The woman (described above in the Privacy section) who was concerned about insurance rate increases believed her insurer learned about its patients through the billing history for medications, and was confident the insurer would not know she had diabetes as long as she continued to avoid the need for medications by controlling her diabetes through diet.

Physicians often recognized that the patient’s understanding of the health care system influenced the way that they shared medical records. Several reported that patients attempting to bring medical records to their doctor mistakenly brought hospital bills or even generic patient information printouts.

### Theme C: Methods And Tools For Information Sharing

The patients who actively managed their own medical information had a variety of strategies for doing so, all of which were described by both patients and providers.

#### Memorization

Some kept track of lab values in their head. Most of the patients with diabetes were accustomed to giving an oral report to the primary care provider about their most recent podiatry, ophthalmology, and dental visits; only rarely were records from these visits transferred. Many informants were confident that they had memorized their medication lists. “I keep it in my head. I drink more than 20 medicines daily, and I know the dosage, the day, for what is this medicine and how many times I drink daily, how many dose.” (Many of the Spanish-speaking informants used the phrase “drink medicine”, even for pills and tablets, as the English translation of the Spanish expression “tomar la medicina”.)

#### Personal Electronic or Paper Records

**Figure 1:**
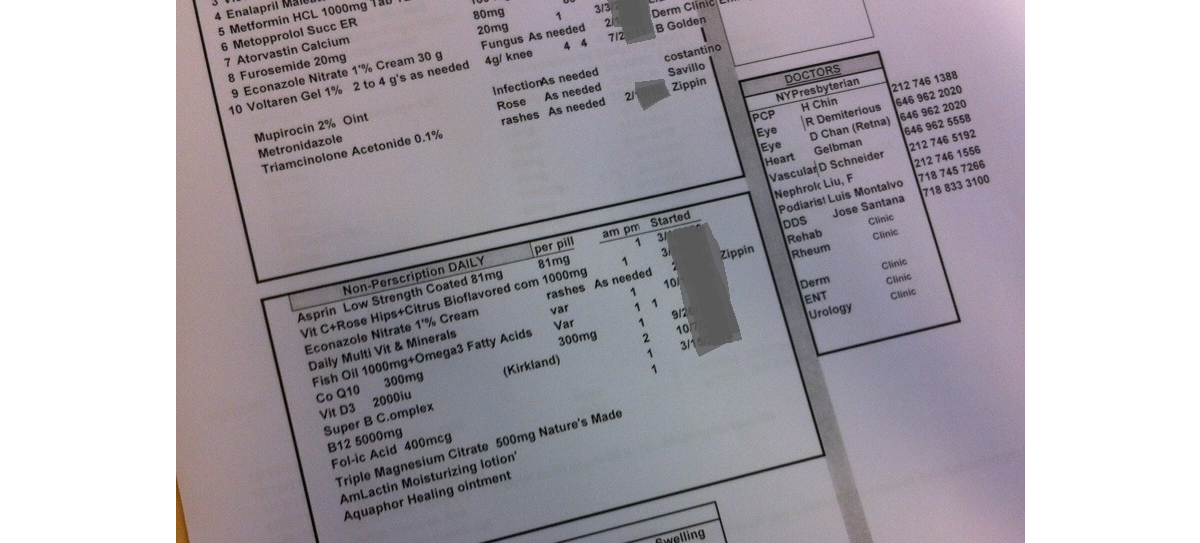
Portion of a 3-page personally tracked record by an individual with multiple chronic diseases. This patient regularly updated the Excel spreadsheet with medications, dates of medical appointments and events, contact information, etc. Dates have been masked.

**Figure 2:**
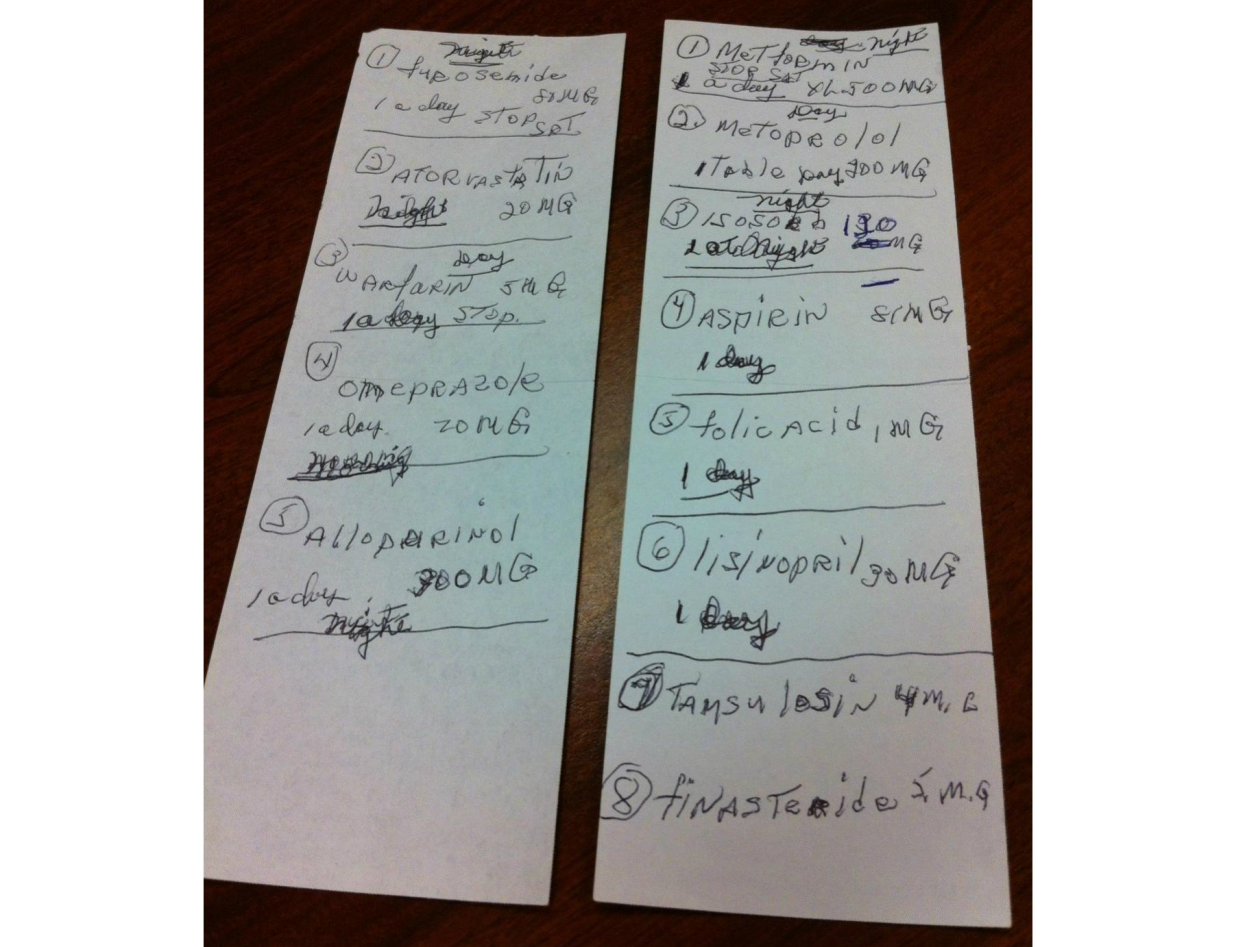
Portion of medication list used by a patient to track 13 medications. Originally, he had designed his system so all daytime medications were on one sheet and all nighttime medications on the other, but as the medication regimen changed, he updated his notations. The patient kept the lists in a plastic grocery bag which he brought to medical appointments.

Keeping or developing paper or electronic documents was less common. Some kept folders containing medical bills, reports, and test results. Two individuals created detailed spreadsheets of past medical history, current medications, physician and personal contact information, recent lab results, and other information which they regularly updated and carried with them (Figure 1). Others used handwritten lists (Figure 2). One who primarily relied on memorization of his medication list used a paper list as a backup. “Sometimes if you’re sick with... pain, the memory don’t work the same.”

#### Original Artifacts

Some patients kept track of information about themselves and their providers by saving objects provided by the health care system, including business cards and empty pill bottles with prescription labels.

#### Electronic Patient Portals

A very small number of patients had experience with patient portals. One had separate portal accounts with his outpatient physician, his hospital, and the Veterans Administration, and used them to help inform his doctors about what went on in the other health care systems (for example, requesting that a colonoscopy report be sent to his primary care physician). One patient used her account to print her medication list for a surgical admission. Although familiarity with computers was more common among younger patients, we observed cases of older, well-educated patients using computers easily and younger patients in less affluent circumstances being unfamiliar with computers.

### Theme D: Managing Medical Information As “Invisible Work”

When patients tracked clinical data such as their own blood pressure, weight, blood glucose, or medication administration, their work was apparent and therefore visible to their doctors and nurses. By contrast, the work they performed to manage records or correct their information was generally invisible to their health care providers. This invisibility raised new challenges: patients found these tasks interfering with their regular illness work and felt they had nowhere to turn for assistance. “Nobody wants to help you,” said one. Medical providers were sometimes aware, in general terms, of the challenges these tasks posed to by their patients, but often found out about the problems long after the patient had already put in substantial effort. Real-time assistance, when it was available, came from people outside the health care system who had previous experience with some of these problems, such as family members or pastors.

The most common event triggering invisible PHIM work was an error in information. Two patients had similar stories about pharmacies substituting their prescription for an extended-release equivalent that their physician had previously decided was inappropriate for them. Other examples included erroneous information in the medical record discovered through the electronic patient portal, important laboratory results missing prior to surgery, delivery of home medical equipment without instructions for use, and mistaken denial of insurance coverage and errors about co-pays and deductibles.

These incidents launched patients into lengthy projects to find relevant information, often accompanied by a search for the correct party to whom to deliver it. Frequently, multiple attempts were needed to resolve the problem. For example, the man who was trying to correct information in his electronic patient portal account got referred from a technical support phone line to the doctor’s front desk staff to a technical support email address and then back again without getting the problem resolved. Another patient recounted: “So my primary doctor did an authorization for it, and I just got the letter that they denied me. Now they’re saying in the letter that was because she didn’t put enough information. So on the 12th I have to go to her with the letter and then I got to ask her what is the information that I need that has to comply with what they’re asking for.” One man seeking instructions for using his medical device said different offices had referred him to different places: “They keep giving you the runaround.”

Several patients developed preemptive procedures to deal with what they expected would be errors. One man said with exasperation that he double-checked the status of every lab result after a situation in which lab tests required prior to his surgery had been lost. “I would follow up… Every time. Not just once. Every time.” Another said he called his insurer before trying to fill any new prescription. “I say, ‘I’m getting this, this, this, this, and this medicine. You cover? You sure? [Give] me your name,’ I say, and I write down the name. And when I had a problem, I called to insurance and I say, ‘somebody with this name gave me this information. Why [with] you now it’s different?’” A third said he routinely stockpiled extra pills before getting a refill because he expected to encounter mistakes about his co-pay, and the extras would give him time to sort out the error before he ran out of medicine.

This work frequently felt frustrating, exhausting, and unfair. Many of our participants became angry when discussing it. Another said it made her “so tired,” and another began crying. One woman dealing with an insurance denial said, “Sometimes I would like to hear a human voice that will be able to reassure me and tell me this is what’s going on.” One woman trying to resolve a disagreement with a home health agency said, “I couldn’t sleep one single minute yesterday.”

One man explained why he had not followed up on a potential route to get insurance coverage for the shingles vaccine. “Who wants to go through all that? Who has the time and energy to continue the struggle, especially someone who is chronically trying to deal with everything else they’ve got to deal with? … It’s hard enough when you’re healthy and you’re with it, and you’re feeling good… When you’re not feeling well at all, it’s difficult. I don’t have the energy. I don’t have the time. I don’t feel good. I don’t want to deal with it.” The same man later said, “It’s hard to be on top of everything. I mean I’m not a computer. I’m a person, you know.”

Unfairness was a frequent theme. A woman seeking to renew a medication for hepatitis C said, “I shouldn’t have to be the one who straightens it out with the insurance company, because that’s their job.… It’s a lot of my time that I’d rather spend with other things.” The unfairness could be linked to the power difference between the insurer who had access to resources and information and the patient who did not. “They just send a [denial] letter and you’re stuck with the rest of the mess when you’re not even familiar with the plan.”

## Discussion

### Principal Findings

Patients with multiple chronic conditions have relationships with complex and changing networks of physicians and other care providers, pharmacies, allied health providers, and insurers. Providers consider it essential that information flows freely across institutional boundaries to help them take care of their patients. Some patients with MCC rely heavily on the health care system itself to maintain up-to-date records and make sure relevant information is accessible to health care professionals who might need it. Yet many other patients take an extremely active role in collecting, monitoring, and transferring their medical records across organizational settings. These patients use a variety of tools and methods to accomplish these tasks, ranging from memorization to requesting documents to using electronic patient portals.

We find that some patients were concerned about the privacy of their medical information because of ways it could be, or had been, used against them. However, privacy concerns came up relatively rarely in our interviews. Instead, patients’ choices about what information to collect and share are strongly shaped by their understanding of health and disease and what information was relevant for specific medical conditions. Medical information is often left out because the patient did not see its relevance (for example, a patient who judged that her dermatology treatment was not relevant to her primary care). Previous work on mental models of disease [39-41] demonstrates that patients may create multiple internally coherent representations (or mental models) of the same disease, and these representations have varying degrees of similarity or difference from the biomedical model promoted by their physicians and nurses. We also find that patients’ decisions about records management were also influenced by their understanding of how the health care system worked. For example, patients did not request records transfers between institutions if they thought doctors at each institution could access the other’s records, and at least one was making medical decisions in part on the basis of whether they would reveal information to her insurer.

We also find that one of the biggest issues facing patients is the enormous amount of work involved in fixing errors, many of which arise from the complexities of seeking care across different institutions or, even more frequently, from complexities in health insurance. This work can be exhausting, upsetting, and frustrating, especially in light of the demands patients already face because of their illness work. Because this work is conducted outside of the relationship with any individual health care provider, it is often invisible to providers.

### Limitations

Our sampling approach focused on English-speaking patients with multiple chronic conditions who were in regular medical care in a major urban area in the United States. The resulting sample was economically diverse but contained few advanced users of information technology. This may put some limits on generalizability to rural patients, people of other cultures, or more experienced users of information technology. Many of the informational challenges reported by our patients arose from negotiating the interface between health care organizations and health insurance companies, and results may not be fully generalizable to the patients of very different health care delivery models, such as integrated delivery systems in the United States or national health care systems in other countries. However, a member of our research team (HOW) found that these themes resonated with her similar experiences in two Canadian provinces. Attending the diabetes education group for ongoing triangulation and relying on the group for the member check could have made the final themes more representative of patients with diabetes than of patients with other chronic conditions. Our focus on information being used by patients in their interactions with the medical system also means that the types of medical information being discussed was probably narrower than the broader range described in some other PHIM literature [22,24,26].

### Comparison With Prior Research

Our findings are highly congruent with perspectives from the sociology of illness. In their landmark 1985 work, Corbin and Strauss described the experience of being diagnosed with chronic disease as ushering in a series of new tasks and responsibilities as illness work [18,19]. Corbin and Strauss focused on activities such as following medication regimens and using home medical equipment, and others have since extended this concept to include the work of managing personal health information [18,19,22,27,28].

Our work contributes to a growing body of work on personal health information management or PHIM [22-25]. To date, much of this work has been performed with generally healthy individuals and families and in cancer. In extending this research to patients with multiple chronic disease, we found many similarities. For example, we found that individuals use both custom-made tools (such as electronic patient portals) as well as paper and pencil and a variety of other artifacts. Others have noted that patients use commercial calendars to track medical appointments, post medication checklists on refrigerator doors, or intermingle pediatric immunization records with memorabilia about the child’s milestones [22,24,26].

However, in many other ways our patients provided a different perspective on PHIM. An earlier study of generally healthy individuals showed that many people rejected the idea that activities such as sharing health records or investigating medical options was *work*, and instead preferred terms such as management [30]. By contrast, one of the most striking findings from our interviews is that those with MCC see many of these activities as work. In particular, our patients had frequent experience with addressing informational errors within and across health care institutions, triggering tasks that were effortful, time-consuming, and emotionally draining, especially in light of the burdens of their existing illnesses. Unruh and Pratt [42] described very active patient work in detecting, preventing, and recovering from medical errors in outpatient cancer treatment. By contrast, our focus on outpatient chronic care meant that most of the errors described by our patients were information errors (such as the failure to transfer laboratory results to a surgeon, leading to the postponement of surgery). These sorts of information errors rarely involved medical errors, although they certainly appeared to have the potential to trigger medical errors.

We propose that these types of work fall in the category of *invisible work*. *Visible work,* such as care provided by doctors and nurses, is recognized, valued, and sometimes compensated. Other examples of *visible work* are informational tasks directly related to disease management that patients take on in collaboration with their providers, such as tracking blood pressure, blood glucose, or diet. By contrast, the concept of *invisible work* [29,30] describes necessary tasks that go unrecognized because they take place outside of the public sphere, require a degree of effort that may not be fully understood by others, or are conducted by people who are not seen as important [43]. Our patients describe tasks that are invisible because they take place almost entirely in the spaces between institutions, such as between health care providers and insurance companies. Health care providers may hear about this work from their patients, or even assist by (for example) providing letters to combat insurance denials. But they are not involved in the day-to-day “struggle” (in the words of one patient) and may hear about these challenges only after patients have already put in considerable time and effort trying to resolve them.

Consistent with what has been found previously by others, our informants were often selective in deciding when and with whom to share medical information [44]. Privacy concerns, although a factor, did not appear to be the most important issue raised. Instead, we find that many patients base these judgments on their own understanding of both health care organizations and disease processes, which may not coincide with their providers’ views.

Although very few of our participants had ever used electronic patient portals, our findings are highly relevant to this rapidly evolving field. Portals are being offered by more and more health care organizations seeking to comply with the “meaningful use” regulations. California Health care Foundation/National Partnership for Women and Families find sharply increasing interest in and access to electronic health records via patient portals, with an estimated half of Americans having access to their electronic records via portals as of 2014 [45,46]. Patients newly exposed to the concept of the patient portal may be enthusiastic about its potential [47]. Those who already have portal access report that it helps them share data with health care providers, find and correct errors in medical records, and avoid having to fill out the same forms repeatedly, which were some of the common tasks mentioned by patients in our study [45,46]. Experiments with sharing the entire medical record (including often-hidden elements such as notes) have had positive results, with many patients feeling empowered and better informed about their care [48,49]. Nevertheless, some patients have reported negative feelings about seeing their electronic records, such as their diagnosis information or their lab results [50]. To date, few high-quality controlled studies have been conducted to assess the effects of portal-based interventions, and relatively few of these have reported positive findings on patient outcomes [15].

### Conclusions and Implications

Because of their complex medical situations, patients with multiple chronic conditions maintain relationships with multiple health care providers, usually spanning several medical institutions. Providers, and many patients, recognize the need for easy information flow across these medical settings. In the absence of seamless health information exchange processes or technologies, patients and providers use a wide variety of workaround approaches, sharing information through combinations of memorization, paper, fax, and electronic tools. Privacy concerns were not universal, but a minority of patients had serious concerns about the ways their medical information might be used. Perhaps more importantly, patients made decisions on the basis of their mental model of their health and disease, providing information that they believed was relevant and omitting other facts that they considered irrelevant. Patients also made decisions on the basis of their understanding of the health care system, including the way that insurance works. To the extent that their mental models fail to coincide with their health care providers’ models, this may lead to significant information gaps or suboptimal decisions. One of the biggest issues facing patients is the enormous amount of difficult, frustrating, and emotionally tiring work involved in addressing informational errors. Because this work is conducted outside of the relationship with any individual health care provider, it is often invisible to their health care providers. Furthermore, because this invisible work arises from complexities in medical care and medical coverage, it seems likely to fall most heavily on those with the most encounters with the medical system, constituting a systemically regressive tax on illness.

Effective structural solutions for information sharing are likely to not only improve the quality of information shared but also reduce the burden on patients already weighed down by MCC. Types of information technologies that might help resolve these problems include health information exchange (HIE) and personal health records (PHRs). Traditionally, HIE technologies are provider-centered, allowing doctors to look up communitywide data on their patient or push an individual patient record to a fellow physician [7,51,52]. By contrast, PHRs are designed for patients to keep and manage their own medical information in electronic form, accessible on the Web or mobile devices [53-55]. In the United States, the “meaningful use” regulations [9] are promoting adoption of “tethered” PHRs, which allow patients to view or export their medical records from a single institution [12]. Tethered PHRs are now routinely offered by many US health insurance companies as well, giving patients access to their insurance claims information and supporting patient education materials. By contrast, “untethered” PHRs give patients full control over collecting, tracking, annotating, and sharing data from multiple institutions or information of their own [56]. Examples include Microsoft HealthVault and Google Health (discontinued in 2011 for lack of adoption.)

Our findings suggest that both provider-centered and patient-centered information technologies will continue to be needed. Each has the potential to support patients in many of the most problematic aspects of health information management, but neither is likely to resolve all problems. Electronic PHRs are likely to hold the most appeal for patients who already take active roles in collecting, managing, and sharing medical information across their fragmented networks of care. These tools are gaining traction [14,21], and yet many still pose barriers related to less than optimal usability, lack of patient-centeredness in both vocabulary and functionality, and lack of integration with devices [57,58]. Furthermore, tethered PHRs offer access only to one institution’s data. They can be enormously helpful for exporting medical records or findings such as lab results, but this alone cannot address all of the between-institution informational gaps that arose in our interviews. In addition, it is critical to acknowledge that, as we found, many patients do not take an active role in managing their own information and even those patients who did manage their information sharing tended to choose what to share based upon lay mental models of health and health care. We and others have also previously found that patients are broadly supportive of provider-facing HIE technologies [20,59]. For all these reasons, it seems likely that patient-centered and provider-centered approaches should be considered complementary, fulfilling different functions for different stakeholder groups. Ultimately, an ideal health information management technology would allow patient data to flow easily across organizational boundaries and also be fully accessible to that subset of patients who wish to view or manage their data [60].

## Abbreviations

HIE: health information exchange
HIT: health information technology
MCC: multiple chronic conditions
PHIM: personal health information management
PHR: personal health record
